# Exploring the Structure of Sibling Relationships Among Preschool Children in China and Developing a Questionnaire

**DOI:** 10.3389/fpsyg.2021.745165

**Published:** 2022-01-17

**Authors:** Meiru Jiang, Xiaojun Cao, Qinqin Huang, Siqi Wu, Xu Chen

**Affiliations:** ^1^Department of Education, China West Normal University, Nanchong, China; ^2^Department of Psychology, Southwest University, Chongqing, China

**Keywords:** preschool children, sibling relationship, questionnaire development, reliability and validity, China

## Abstract

**Objective:**

The objective of this study was to examine the structure of sibling relationships among preschool children in China and develop a questionnaire.

**Methods:**

The concept of sibling relationships among preschool children in China was established through literature review, open interviews, and expert review, and the initial project was designed. Using the questionnaire survey method, with 651 mothers of preschool children as the research objects, we performed item analysis, exploratory factor analysis, confirmatory factor analysis, and reliability and validity tests on the initial questionnaire.

**Results:**

The questionnaire for the sibling relationships among preschool children in China had 18 questions covering dimensions of sibling interaction, sibling acceptance, sibling warmth, and sibling rivalry. The questionnaire fitting indexes were better (χ^2^/*df* = 1.829, CFI = 0.930, TLI = 0.917, RMSEA = 0.055, SRMR = 0.053). The internal consistency coefficient between the total questionnaire and each dimension was 0.759–0.8548, and the total questionnaire significantly correlated with each dimension (*r* = 0.229–0.792) and the total score of parental self-efficacy (*r* = –0.106 to 0.338).

**Conclusion:**

The Sibling Relationship Questionnaire for Chinese Preschool Children (Parental Version) exhibited good reliability and validity, making it an effective tool for the evaluation of sibling relationships among preschool children.

## Introduction

The sibling relationship signifies the psychological and emotional relationships among children in a nuclear family through interaction and influence (Bekkhus et al., 2016). The sibling relationship is one of the most crucial and lasting relationships in the life of a person and significantly contributes to social, emotional, and psychological development in children (Wong et al., 2010). Previous research has often focused on establishing a connection between a sibling relationship and negative development (attack, crime, and blocked executive function) of children (Mei et al., 2009; Buist et al., 2019). In addition, the “overflow hypothesis” validates that a negative sibling relationship not only affects the development of a child but also affects other systems, as well as adversely affects peer relationships and parent-child relationships (Ruff et al., 2018). Besides, adverse interactions among siblings of preschool children can predict internalizing and externalizing behaviors of teenagers. With the intensification of research and the perfection of theory, the positive impact of the sibling relationship has also become a hot topic of research. Previously published studies have demonstrated that sibling warmth (SW) can accelerate the development of the prosocial behaviors of children (e.g., help, comfort, and share) (Hughes et al., 2018) and play a vital role in the adaptation and happiness of children and adolescents (Piotrowskia et al., 2014). The development and effects of sibling relationships are not only dynamic but also lasting. A follow-up study illustrated that early relationship between siblings of different genders is a significant factor influencing the adolescent happiness and dating ability of an individual (Doughty et al., 2015). The closer the relationship with siblings of different genders is, the stronger are the happiness and ability to love in adolescence. Conversely, the more frequent the conflict with siblings of different genders in early childhood, the lower the happiness and love ability. The simple stacking of siblings does not necessarily significantly affect the social development of children, while the quality of the sibling relationship is a critical influencing indicator. In summary, a positive sibling relationship is of great significance for the social development of children and teenagers and is a protective factor in handling external risks. However, a negative sibling relationship often leads to various internalizing and externalizing behaviors of children and teenagers and is the risk factor that affects their social development (Dirks et al., 2015). Furthermore, a sibling relationship plays a crucial role in the growth of children regardless of being a risk factor or a protective factor.

The research on sibling relationships is relatively slow in China due to the long-term birth control policy (Chen and Shi, 2017). In 1978, the “one-child” policy was enshrined in the constitution, which mandated couples to have only one child. The birth control policy was implemented for more than 30 years to ensure that the population growth was consistent with economic and social development plans. In 2013, the Third Plenary Session of the 18th Central Committee officially opened the “separate second child,” that is, a couple could have a second child if one of them were an only child. From 2015, the Fifth Plenary Session of the 18th Central Committee further decided to fully implement the “comprehensive second child” policy where a couple could have two children, which marked the complete end of the one-child policy in China implemented for over 30 years. With the changes in childbirth policy, more children and teenagers in China would enter the sibling relationship from being the only child. During this period, “whether to have a second child” and “how to deal with a sibling relationship” have become hot topics in the current Chinese society (Chen et al., 2016).

Currently, the conventional tools for measuring sibling relationships in China are primarily questionnaires principally compiled by foreign scholars, and most of them focus on measuring the quality of sibling relationships from late childhood to adolescence (Francka et al., 2019). Of these, the most classic is the Sibling Relationship Questionnaire (SRQ) compiled by Furman and Kramer’s Parental Expectations and Perceptions of Children’s Sibling Relationships Questionnaire (PEPC-SRQ). The Sibling Relationship Scale compiled by Furman uses self-reporting to collect data and categorizes sibling relationships into four dimensions, namely, intimacy, conflict, competition, and power comparison (Furman and Buhrmester, 1985); notably, this is the most used tool to measure the sibling relationship of teenagers in China. The “PEPC-SRQ,” compiled by Kramer for children in early childhood, uses the method of parental reporting to collect data and categorizes sibling relationships into three dimensions, namely, SW, sibling rivalry (SR), and competitive motivational behaviors (Kramer and Baron, 1995). From the viewpoint of cross-cultural research, due to the differences in the concept of familyism between Eastern and Western cultures, there are differences in the structure and characteristics of sibling relationships from different cultural backgrounds (Rowan, 2016). This study aims to take the firstborn preschool children as the research object to investigate the structure of sibling relationships among preschool children under the Chinese cultural background, to provide a reliable tool for measuring early sibling relationships.

## Materials and Methods

### Subjects

Mothers of firstborn children (age 3–6 years) from five kindergartens in Chengdu, three in Nanchong, and one in Xichang were selected as research objects. First, 30 mothers of firstborn children were invited to participate in an open interview. Second, 617 mothers of firstborns were given questionnaires at various stages of the study. Finally, a total of 591 valid questionnaires were obtained after removing the questionnaires with irregular responses, multiple-choice, and missing-choice. The questionnaires were subjected to exploratory factor analysis, confirmatory factor analysis, and reliability and validity tests using four independent samples.

Sample 1: Open interview with questionnaire dimensions and project preparation, with a total of 30 people (15 boys and 15 girls);
Sample 2: Item analysis and exploratory factor analysis, with a total of 156 people (72 boys and 84 girls);
Sample 3: Confirmatory factor analysis, with a total of 271 people (130 boys and 141 girls);
Sample 4: School standard validity analysis, with a total of 164 people (88 boys and 76 girls).

### Development of an Initial Questionnaire

The sibling relationship is multidimensional, and it comprises positive and negative sibling relationships (Howe et al., 2011). In this study, sibling relationship among preschool children was measured from the perspective of early childhood, based on the characteristics of sibling interaction (SI) of preschool children and focusing on the three aspects of the composition of the sibling relationship, the questionnaire report method, and the evaluation subject. First, regarding the questionnaire report method, parental reporting was used to collect data based on the cognitive characteristics of preschool children, thereby evading the inconvenience of self-reporting. Second, regarding the questionnaire evaluation, previous studies have established that the imbalance of rights between siblings is most noticeable in early childhood. During this phase, the behavior of firstborn children in sibling relationships is more dominant, more stable, and predictive (Kolak and Volling, 2011). Thus, taking the behavior of firstborn children between siblings as the subject, we compiled the Sibling Relationship Questionnaire for Chinese Preschool Children (Parental Version).

Using a combination of literature and interview methods and through the theoretical analysis, literature review, and interviews with subjects and experts, we constructed the initial measurement structure of sibling relationships among preschool children. In the first step, the researcher and two postgraduates majoring in preschool education conducted open interviews with mothers of 30 firstborn children, as well as guided them to describe and record positive and negative interaction events between siblings in daily life, including “Will your Dabao help Erbao do something? What to do?” In the second step, five first-line kindergarten teachers with >3 years of teaching experience were invited to supplement and delete the original interview data. In the third step, 1 psychology professor, 1 preschool education associate professor, and 12 postgraduates majoring in preschool education were invited to rewrite, merge, classify, and process the first-hand information collected from the interviews to attain the most frequent daily events that best reflect the sibling relationship, as well as determine the dimensions and questions of the initial version of the Sibling Relationship Questionnaire for Chinese Preschool Children (Parental Version). The initial questionnaire consisted of 35 questions, including SI, sibling acceptance (SA), SW, and SR. The questionnaire was in Mandarin, which is the predominant language in China, and used a 5-point Likert scale for evaluation (with a range of 1 = “never happen” and 5 = “always happen”).

### Data Processing

We used IBM SPSS STATISTICS 23.0 and Mplus7.0 to manage and analyze the data in this study.

## Results and Analysis

### Item Analysis

To test the rationality of the question settings and the structure of the questionnaire, we performed item analysis on the initial questionnaire. First, the critical ratio (CR) between the high and low groups was used to estimate the question discrimination. Then, the total score of the sample was categorized into the high and low groups, with 27% as the critical point. In addition, an independent-sample *t*-test was performed to evaluate the difference between the two groups and the average of each question, as well as validate whether each question fulfills the requirements of statistical difference. Of note, the questions with CR < 3 were deleted. Then, based on the correlation between the question and the total score of the questionnaire, we tested the degree of discrimination of the questions and deleted the questions with low correlation (*r* < 0.40). Finally, there was a deletion of four questions in the item analysis, while the remaining 31 questions entered the exploratory factor analysis.

### Exploratory Factor Analysis

The maximum likelihood method was used to perform the exploratory factor analysis on the initial test data of sibling relationships of preschool children. Then, common factors were extracted from it, and the final factor load matrix was attained using the orthogonal rotation method. After several explorations, we deleted seven questions with the cross-load and low load (<0.4) and performed the second exploratory factor analysis on the remaining 24 questions.

**FIGURE 1:**
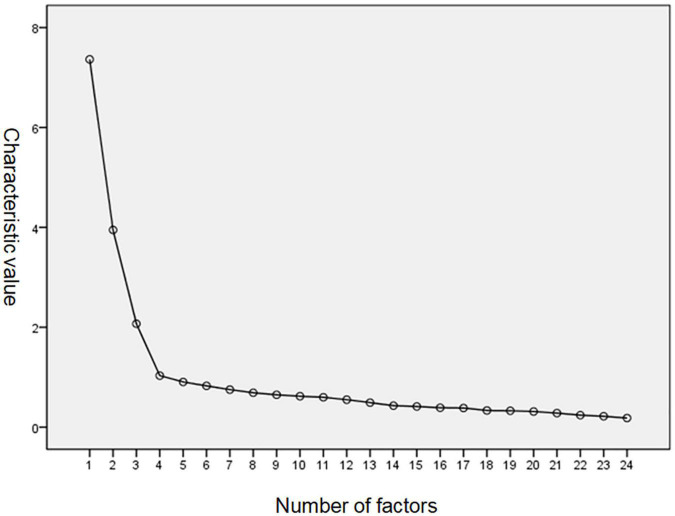
The screen plot.

Through KMO and Bartlett’s test, KMO was 0.883, indicating that the data had a strong partial correlation, and the impact of factor analysis was very good. In addition, the χ^2^ value of Bartlett’s sphere test was 2824.527 (*df* = 276, *P* < 0.001) and was significant. Hence, the assumption that each variable is independent should be rejected; that is, the variables are strongly correlated, and the data are suitable for factor analysis. Under the condition of not limiting the number of factors, we used the principal component method to extract four factors with characteristic roots >1. The explanation amount was 52.27%. Meanwhile, the principal component characteristic scree plot indicated that the eigenvalues of the first four factors had a steep decline. The eigenvalue distribution tended to be flat from the fifth factor, and each factor contributed less to the accrued explained total variation. Overall, it is more reasonable for sibling relationships among preschool children to use four structural factors. Figure 1 shows the scree plot.

The results of the exploratory factor analysis were roughly the same as the expected dimensions. Hence, the four factors were named as SI, SA, SR, and SW. SI contained seven questions with the factor load of 0.649–0.777; SA contained six questions with the factor load of 0.608–0.784; SR contained six questions with the factor load of 0.655–0.829, and SW contained five questions with the factor load of 0.674–0.763. Table 1 shows the factor loading matrix of the question.

**TABLE 1:** The factor loading matrix of the questionnaire.

Number	Question	Sibling interaction (SI)	Sibling acceptance (SA)	Sibling rivalry (SR)	Sibling warmth (SW)	Deleted items
1	Dabao and his younger brother/sister participate in games, crafts and planting activities together	0.777				
	大宝和弟弟/妹妹会一起参加游戏、手工和种植等活动					
2	Dabao and his brother/sister tell each other their little secrets	0.763				
	大宝和弟弟/妹妹会相互告诉对方自己的小秘密					
3	Dabao would bring his younger brother/sister along when playing with other companions	0.74				
	和其他同伴玩耍时，大宝会带上弟弟/妹妹一起					
4	Dabao and his brother/sister keep each other’s little secrets	0.72				Deleted
	大宝和弟弟/妹妹会保守彼此的小秘密					
5	Dabao will teach his younger brother/sister what he learned in kindergarten	0.676				
	大宝会把在幼儿园学到的知识教给弟弟/妹妹					
6	Dabao will tell his brother/sister the interesting things that happened in kindergarten	0.672				
	大宝会把幼儿园发生的有趣事情讲给弟弟/妹妹听					
7	Will Dabao teach your younger brother/sister how to dress and eat	0.649				
	大宝会教弟弟/妹妹如何穿衣吃饭					
8	Dabao will show off his brother/sister to others		0.784			
	大宝会在别人面前炫耀自己有弟弟/妹妹					
9	Dabao will be proud of having a younger brother/sister		0.765			
	大宝会因为自己有弟弟/妹妹而感到骄傲					
10	Dabao will say things like “I love my brother/sister”		0.69			
	大宝会说“我喜欢弟弟/妹妹”之类的话					
11	Dabao often talks about his younger brother/sister in front of others		0.681			
	大宝会在别人面前经常提起自己的弟弟/妹妹					
12	Dabao is looking forward to or happy about the birth of his younger brother/sister		0.654			
	大宝对弟弟/妹妹的出生感到期待或高兴					
13	Dabao will not allow others to speak ill of his brother/sister		0.608			Deleted
	大宝不允许别人说弟弟/妹妹的坏话					
14	Dabao will often control the younger siblings, do not touch their own things and so on			0.829		
	大宝会经常管制弟弟妹妹，不准碰自己的东西等					
15	Dabao gets angry when his brother/sister breaks his things			0.795		
	大宝会因为弟弟/妹妹弄坏自己的东西而生气					
16	Dabao and his younger brother/sister would shout and even pull and push each other when arguing			0.777		
	大宝和弟弟/妹妹发生争执时会大吼大叫，甚至拉扯推搡对方					
17	Dabao does not allow his younger brother/sister near his playing objects			0.703		
	大宝不允许弟弟/妹妹靠近自己玩耍的物品					
18	Dabao and his younger brother/sister would argue with each other			0.685		Deleted
	大宝和弟弟/妹妹发生争执时会互不相让、争论不休					
19	When the younger brother/sister is in trouble, Dabao will ask the reason and solve the younger brother/sister’s problem			0.655		
	弟弟/妹妹遇到困难时，大宝会询问原因并解决弟弟/妹妹的难题					
20	When your younger brother/sister is sad, Dabao will ask each other why they are crying and coax them				0.763	
	大宝会在弟弟/妹妹伤心时询问对方为什么哭，并哄他们					
21	Dabao will protect his/her younger brother/sister when they are afraid				0.739	
	大宝会在弟弟/妹妹感到害怕时保护他们					
22	When the younger brother/sister is in trouble, Dabao will ask the reason and solve the younger brother/sister’s problem				0.697	
	弟弟/妹妹遇到困难时，大宝会询问原因并解决弟弟/妹妹的难题					
23	Dabao will touch, pat and hug his/her younger brother/sister when they are sad				0.692	
	大宝会在弟弟/妹妹伤心时抚摸、轻拍、拥抱弟弟妹妹					
24	Dabao will cheer up his younger brother/sister with snacks or toys when they are sad				0.674	Deleted
	大宝会在弟弟/妹妹伤心时，用零食或玩具哄他们开心					

*The marked questions have been removed from the final post-CFA questionnaire.*

### Confirmatory Factor Analysis

**FIGURE 2:**
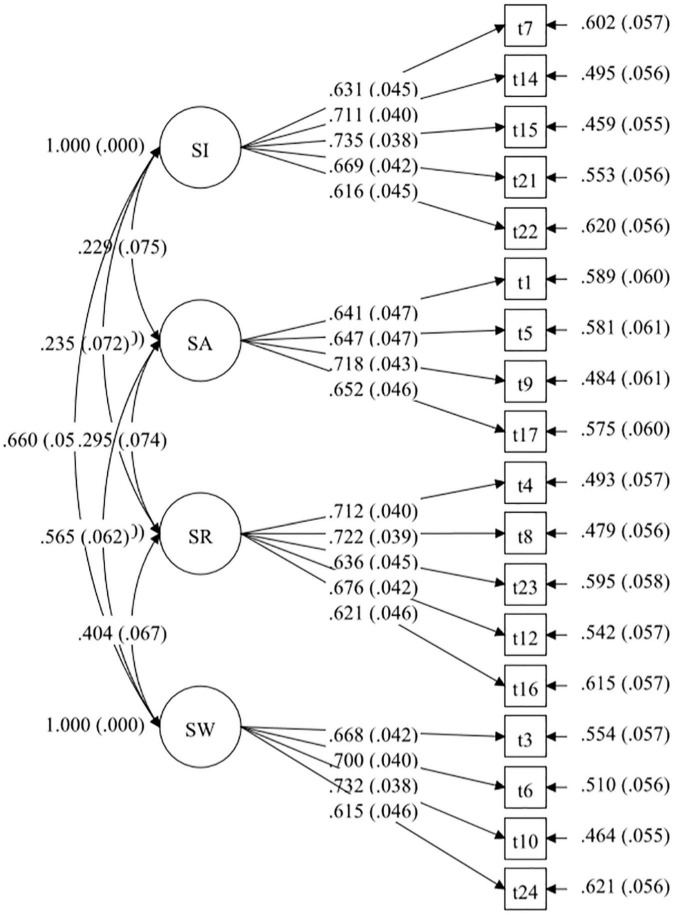
Simulation diagram of the model path.

Based on the variance-covariance matrix of 24 items in the questionnaire for sibling relationships of preschool children, we used the maximum likelihood method to perform the confirmatory factor analysis on the conceived four-factor model. Figure 2 shows the path of the model.

In general, the model is better when the value of χ^2^ is smaller and the value of df is larger. Of note, χ^2^/*df* < 5 indicates that the model is acceptable, while χ^2^/*df* < 3 indicates that the model is better (Cox et al., 2002). In addition, the values of RMSEA and SRMR < 0.08 indicate that the model is acceptable (Kline, 2011), while the values of CFI and TLI should be > 0.9 and closer to 1 (Byrne, 2011). The individual indicators were not ideal in the initial fitting test of the model. After removing the higher cross-loading questions based on the modified index, the secondary confirmatory factor analysis was performed, and all fitting indexes of the model reached the ideal value. The fitting indexes of the questionnaire model were as follows: χ^2^/*df* = 1.829, CFI = 0.930, TLI = 0.917, SRMR = 0.053, RMSEA = 0.055. As observed, the value of χ^2^/df of the revised questionnaire was 1–3, and the SRMR and RMSEA were < 0.08, and the CFI and TLI were both > 0.9, suggesting that the model has a good degree of fit. A total of six questions were deleted in the confirmatory factor analysis (refer to Table 1). A total of 18 questions were finally retained to form the formal Questionnaire for Sibling Relationship of Preschool Children. Table 2 illustrates the analysis of the degree of fit of the model.

**TABLE 2:** Analysis of the degree of fitting of the model.

Fitness index	Key value (recommended value)	Model indicators before revision	Model indicators after revision	Correction result
χ^2^	The smaller the better	464.848	235.938	
df	The larger the better	224.000	129.000	
χ^2^/df	1 < χ^2^/*df* < 3	2.075	1.829	Yes
CFI	>0.9	0.891	0.930	Yes
TLI	>0.9	0.877	0.917	Yes
RMSEA	<0.08	0.063	0.055	Yes
SRMR	<0.08	0.062	0.053	Yes

### Validity Analysis

#### Convergent Validity

To confirm the validity of the questionnaire, a validity analysis was performed on the formal questionnaire. The analysis results indicated that the standardized factor loadings of the 18 questions were all >0.6, suggesting that the dimensions had a strong ability to explain the questions. The standardized factor loadings of the five items under the SI dimension were 0.616–0.735, the standardized factor loadings of the four items under the SA dimension were 0.641–0.718, the standardized factor loadings of the five items under the SR dimension were 0.621–0.722, and the standardized factor loadings of the 4 items under the SW dimension was 0.615–0.732. Notably, AVE denotes the convergent validity of each dimension. The convergent validities of SI, SA, SR, and SW were 0.455, 0.443, 0.456, and 0.463, respectively, which were all greater than the acceptable value of 0.36, suggesting that each dimension had a higher average explanatory ability for the questions.

#### Discriminant Validity

From the correlation of questions in each dimension, the correlation between dimensions, and the correlation between dimensions and the total score, we examined the discriminant validity of the questionnaire. The correlation coefficients between dimensions were 0.229–0.660, which moderately correlated and significantly correlated (*P* < 0.01), suggesting that under the premise that the measurement direction is consistent, the dimensions are different and cannot be replaced with each other. The correlation coefficients between each dimension and the total score were 0.648–0.717, showing a high positive correlation and significant correlation (*P* < 0.01), suggesting that the concept of dimensions contained in the questionnaire was consistent. The AVE root sign value is the correlation between the internal questions of each dimension, and the correlation coefficient revealed a higher positive correlation of 0.666–0.680, suggesting that the questions contained in each dimension had a higher consistency. The correlation coefficient between dimensions was markedly smaller than the correlation between questions within the dimensions and correlation between dimensions and the total score, suggesting that the questionnaire has good discriminant validity. Table 3 shows the analysis of convergent validity and discriminant validity of the questionnaire.

**TABLE 3:** Analysis of convergent validity and discriminant validity.

	Factor loading	Convergent validity	Discriminant validity			
Dimension	STD.LOADING	AVE	SI	SA	SR	SW
SI	0.616–0.735	0.455	0.675**			
SA	0.641–0.718	0.443	0.229**	0.666**		
SR	0.621–0.722	0.456	0.235**	0.295**	0.675**	
SW	0.615–0.732	0.463	0.660**	0.565**	0.404**	0.680**
General questionnaire			0.717**	0.688**	0.648**	0.792**

*The bold font on the diagonal represents the AVE root sign value; the lower triangle represents the Pearson’s correlation of the dimension.*

*P < 0.05, **P < 0.01, ***P < 0.001, the same below.

### Reliability Analysis

To test the reliability of the questionnaire, the reliability tests were conducted on the questionnaire and its dimensions, including Cronbach’s internal consistency coefficient, split-half reliability, and test-retest reliability tests. The α coefficient of each dimension was 0.759–0.810. The questionnaire was divided into half according to the odd-even question, and the split-half reliability calculation method was used to attain the split-half reliability of each dimension, which was 0.732–0.787. After a 4-week interval, 86 subjects were selected from Sample 3 for questionnaire retest. The test-retest reliability coefficient *r* of each dimension was 0.821–0.860. All the above-mentioned test results fulfilled the psychometric requirements, suggesting that the questionnaire had high homogeneity and reliability. Table 4 presents the reliability analysis of the questionnaire.

**TABLE 4:** The reliability analysis of the questionnaire.

Questionnaire and its dimensions	The number of questions	Cronbach’s coefficient	Spearman–Brown coefficient	Test–retest reliability *r*
Sibling interaction (SI)	5	0.804	0.787	0.821
Sibling acceptance (SA)	4	0.759	0.749	0.841
Sibling rivalry (SR)	5	0.805	0.766	0.860
Sibling warmth (SW)	4	0.810	0.732	0.840

## Discussion

Based on the PEPC-SRQ structure, this study used a bottom-up structural optimization interview to collect the questionnaire items, aiming to compile the Sibling Relationship Questionnaire for Chinese Preschool Children (Parental Version). Previous studies have reached no consensus on the structure of sibling relationships at different ages (toddlers, children, teenagers, and adults) and different groups (normal groups and special groups). Further analysis revealed that regardless of the division of dimensions, the structure of sibling relationships can be either positive sibling relationships or negative sibling relationships. Kramer divided the sibling relationship into five dimensions, namely, involvement, intimacy, hostility, control, and competition (Kramer and Kowal, 2005); while involvement and intimacy belong to the positive sibling relationship, the latter three belong to the negative sibling relationship. In addition, the three-dimensional division of Campione-Barr is as follows: conflict, trust, and communication (Campione-Barr and Smetana, 2010), which can also be summarized into a positive and negative sibling relationship, where trust and communication belong to positive sibling relationship, while conflict belongs to the negative sibling relationship. Kim et al. (2006) claimed that sibling relationships comprised two dimensions, namely, positive sibling relationship and negative sibling relationship. Thus, using positive and negative sibling relationships as the thread, we developed a local measurement tool for assessing sibling relationships in early childhood of Chinese children through six steps, namely, expert interpretation, item analysis, exploratory factor analysis, confirmatory factor analysis, validity test, and reliability test.

This study indicates that sibling relationship in early childhood has a similar structure and behavior in Chinese and Western cultural backgrounds, that is, sibling relationship includes both positive and negative relationships. However, under the unique cultural background of China, the early sibling relationship of Chinese children is also unique and new. Generally, compared with Western mothers, mothers of Chinese infants reported less sibling competition, such as “Does the mother love, like, and approve Dabao more than her younger siblings?” This could be because specific cultural backgrounds attach different importance to individual independence; Western culture pursues individualism, advocates individual autonomy and independence more, and inspires children to participate in a competition and safeguard individual interests. China, in contrast, has a collectivist culture, emphasizing the interdependent relationship between people and the harmonious relationship between groups, with less encouragement for competition. Thus, in Chinese culture, positive sibling relationship behaviors are more diversified, whereas negative sibling relationship behaviors are primarily sibling conflict and less intense competition and confrontation.

Regarding positive sibling relationships, our questionnaire includes three dimensions of SI, SA, and sibling friendship, while PEPC-SRQ has only one dimension of the positive sibling relationship, namely, sibling intimacy. The most significant difference is that Western children display more direct intimate actions to their siblings, such as “Big baby and brother/sister have a pat, kiss on the cheek, hug, and other intimate actions.” Chinese children, in contrast, find it difficult to comfort their compatriots with a kiss on the cheek. In fact, the implication of collectivist culture is not expressed in fraternal relationships; even on other occasions, they rarely express their feelings *via* kissing (Yau and Lin, 1990). In traditional Chinese concepts, excessive self-expression is often considered a manifestation of poor self-regulation and social immaturity (Ho, 1989). Nevertheless, in an individualistic culture, direct emotional expression and intimate communication are considered greater virtues, contributing to individual self-construction (Rothbaum et al., 2000).

Regarding negative sibling conflict, our questionnaire combined the three dimensions of confrontation, competition, and power compared with PEPC-SRQ, which only included sibling conflict. Regarding sibling conflict, Chinese mothers reported mostly physical conflict, arguing, and grabbing, but Western parents also reported SR over ownership of space (Rothbaum et al., 2000). American culture emphasizes individualism and independent self-construction (Triandis, 1989). Children are typically endowed with the right to enjoy their own space, and the boundary between themselves and others (including significant others) is usually well-defined. However, Chinese culture emphasizes the interdependence between individuals, highlighting the commonality between individuals and group members (Oyserman et al., 2002), and the boundary between self and others is blurred and permeated with each other. Hsu (1981) used a typical family setting as a metaphor to elucidate how Chinese and American cultures construct the self in the family environment. American families often use short fences to separate their homes from their neighbors but set clear boundaries to protect privacy within the home. While Chinese families usually build high-walled courtyards to separate the family from the outside world, there are few clear boundaries to separate the space between family members inside the family.

Overall, the Sibling Relationship Quality Questionnaire for Preschoolers (Parental Version) compiled in this study comprised 18 questions, including four dimensions, namely, SI, SA, sibling friendship (i.e., SW), and sibling conflict (i.e., SR). The test established the ideal reliability and validity of the scale, and all the indicators fulfilled the requirements of psychometrics; hence, it can be used as a reliable tool for measuring the sibling relationship of preschool children in China.

## Data Availability Statement

The original contributions presented in the study are included in the article/supplementary material, further inquiries can be directed to the corresponding author.

## Ethics Statement

The studies involving human participants were reviewed and approved by the Research Ethics Committee of the China West Normal University. Written informed consent to participate in this study was provided by the legal guardian of the participant, and written informed assent was provided by the participant.

## Author Contributions

MJ, XJC, and XC devised the project and designed the study. MJ and QH developed the theory and performed the computations. QH and SW verified the analytical methods. MJ and XC took the lead in writing the manuscript. All authors provided critical feedback and helped shape the research, analysis, and manuscript.

## Author Disclaimer

The content is solely the responsibility of the authors and does not necessarily represent the official views of the various funding organizations.

## Conflict of Interest

The authors declare that the research was conducted in the absence of any commercial or financial relationships that could be construed as a potential conflict of interest.

## Publisher’s Note

All claims expressed in this article are solely those of the authors and do not necessarily represent those of their affiliated organizations, or those of the publisher, the editors and the reviewers. Any product that may be evaluated in this article, or claim that may be made by its manufacturer, is not guaranteed or endorsed by the publisher.
